# Molecular Reclassification of Crohn’s Disease: A Cautionary Note on Population Stratification

**DOI:** 10.1371/journal.pone.0077720

**Published:** 2013-10-17

**Authors:** Bärbel Maus, Camille Jung, Jestinah M. Mahachie John, Jean-Pierre Hugot, Emmanuelle Génin, Kristel Van Steen

**Affiliations:** 1 UMR843, INSERM, Paris, France; 2 Bioinformatics and Modeling, GIGA-R, University of Liège, Liège, Belgium; 3 UMR843, Institut National de la Sante et de la recherche Medicale, Paris, France; 4 Service de Gastroentérologie Pédiatrique, Hôpital Robert Debré, APHP, Paris, France; 5 CRC-CRB, CHI Creteil, Creteil, France; 6 Labex Inflamex, Université Paris Diderot, Paris, France; 7 UMR1078, Génétique, Génomique fonctionnelle et Biotechnologies, INSERM, Brest, France; 8 Centre Hospitalier Régional Universitaire de Brest, Brest, France; Foundation IRCCS Policlinico S. Matteo, Italy

## Abstract

Complex human diseases commonly differ in their phenotypic characteristics, e.g., Crohn’s disease (CD) patients are heterogeneous with regard to disease location and disease extent. The genetic susceptibility to Crohn’s disease is widely acknowledged and has been demonstrated by identification of over 100 CD associated genetic loci. However, relating CD subphenotypes to disease susceptible loci has proven to be a difficult task. In this paper we discuss the use of cluster analysis on genetic markers to identify genetic-based subgroups while taking into account possible confounding by population stratification. We show that it is highly relevant to consider the confounding nature of population stratification in order to avoid that detected clusters are strongly related to population groups instead of disease-specific groups. Therefore, we explain the use of principal components to correct for population stratification while clustering affected individuals into genetic-based subgroups. The principal components are obtained using 30 ancestry informative markers (AIM), and the first two PCs are determined to discriminate between continental origins of the affected individuals. Genotypes on 51 CD associated single nucleotide polymorphisms (SNPs) are used to perform latent class analysis, hierarchical and Partitioning Around Medoids (PAM) cluster analysis within a sample of affected individuals with and without the use of principal components to adjust for population stratification. It is seen that without correction for population stratification clusters seem to be influenced by population stratification while with correction clusters are unrelated to continental origin of individuals.

## Introduction

Many human diseases have a complex genetic architecture and differ in their expression of symptoms. Such phenotypic variability, i.e., variation in the phenotypic expression of one underlying disease, has important consequences for the treatment of affected individuals. For example, Crohn’s disease (CD) patients vary with regard to disease severity, behaviour and location. Classification of inflammatory bowel disease (IBD) cases into similar groups of patients is highly relevant to determine the appropriate mode of therapy delivery and care intensity per patient group [1,2]. So far, existing classification rules for Crohn’s disease have mainly been based on phenotypic clinical measurements [3]. However, under the impulse of the International IBD Genetics Consortium (IIBDGC) and other research groups world-wide, an increasing knowledge about genetic predisposing factors for IBD has become available [4,5]. A meta-analysis of 15 genome-wide association studies (GWAS) and Immunochip data has recently identified 163 loci associated to IBD, of which 30 loci were uniquely associated to Crohn’s disease while 23 loci were ulcerative-colitis-specific [5]. 

Previous efforts to link associated genetic markers with classic clinical CD subphenotypes have detected a clear association between NOD2/CARD15 variants and ileal disease location [6-8]. For other clinical subphenotypes, no robust association with disease susceptibility genes could be shown. This raises the questions of whether more sophisticated and powerful strategies are required to link IBD genetic loci to known clinical subphenotypes, or whether latent (yet to define) patient groups exist that are driven by molecular mechanisms. 

In earlier work of our research group, a first attempt has been made to reclassify CD patients based on single-nucleotide polymorphisms (SNPs) via model-based cluster analysis [9]. Although significantly different genetic-based subgroups could be found, only modest differences between these groups with regard to clinical subphenotypes were described. Our results led to a plea to identify and use molecular information for improved disease risk prediction and disease classification [1], but at the same time highlighted some important issues to consider during the classification process [9]. 

One important issue is the assessment of how disease-specific identified molecular groups are and to what extent these groups overlap with general population strata. It is widely acknowledged that population stratification, which refers to varying allele frequencies between subpopulations in the study population due to different ancestries, is a confounding factor in GWAS leading to spurious association results and failure to detect true associations when not properly accounted for [10]. In this paper, we therefore focus on the effect of population stratification on the classification and on methods to correct for population stratification when clustering affected individuals into genetic-based groups. 

Cluster analysis of individuals based on SNPs has been performed with different purposes, e.g., the detection of genetic-based patient groups, the dissection of trait heterogeneity or the identification of disease susceptible SNPs [9,11-13]. To our knowledge, confounding by population stratification has largely been neglected in any of these analyses. The ignorance of population stratification for cluster analysis of individuals based on genetic data is quite surprising for two reasons.

Firstly for population stratification as for the clustering purposes described above, similar, if not the same, resources (i.e., genetic markers) and analysis methods are used. Indeed, latent class analysis as applied in Cleynen et al. [9] to detect genetic-based subgroups has also been proposed to detect population stratification [14]. Latent class analysis is methodologically similar to the model-based cluster analysis implemented in the software STRUCTURE for investigation of population stratification and can thus be classified as structured association method [9,15]. Other authors have suggested hierarchical clustering and the k-means algorithm to detect population structure which can subsequently be taken into account for association studies [16,17]. 

Secondly, the non-consideration of population stratification is surprising since Myles et al. [18] indicated that some disease risk alleles show an unusual high differentiation between populations to the extreme of being entirely absent in a population. The reason for these strong differences in allele frequencies at certain disease associated markers may be geographically-restricted positive natural selection [18]. Thus, using a set of disease predisposing markers to classify affected individuals into genetic-based subgroups can likely be confounded by population stratification. For example, Myles et al. [18] described that SNP rs10761659, which is associated to Crohn’s disease, gave rise to extremely elevated F_st_ values for pairwise comparisons of African and non-African populations. In other words, an unusual high differentiation in allele frequency between African and non-African populations was observed for this SNP. More subtle differences may also exist in allele frequencies for disease predisposing markers between European populations, e.g., for the SNP rs916977. This SNP is located in the HERC2 gene known to be involved in iris colour and to show population stratification in Europe [19], but the HERC2 gene has also been reported to be associated with Crohn’s disease in a Dutch-Belgian cohort study [20].

In this research paper, we apply several cluster analysis methods on available data for Crohn’s disease to study the complex architecture underlying Crohn’s disease by identifying genetic-based groups. We illustrate the necessity to adjust such a cluster analysis for population substructure and give leads on how to do this with minimal genotyping costs in the absence of a genome-wide SNP panel. In particular, we suggest the use of principal components to correct SNPs for population stratification prior to the cluster analysis. Principal components are commonly used as covariates in logistic or linear regression analysis for genome-wide association studies to handle confounding by population stratification. Similarly to our approach, Zhao et al. [21] regressed the SNPs using principal components to correct for population stratification before performing a random forest analysis. After the cluster analysis, we studied the obtained homogeneous genetic-based patient groups or clusters further to detect association with commonly used clinical subphenotypes. 

## Methods

### Data.

#### Patients .

A total of 845 CD patients were recruited from six paediatric and adult gastroenterology tertiary centres. The diagnosis of IBD was based on Lennard-Jones criteria [22]. Patients were included between April 2002 and October 2003 if the diagnosis of CD was done at least one year before inclusion and if the patients had a continuous follow-up in the reference centres. Patients affected by a disease treated by chemotherapy or radiotherapy were excluded. The study received an ethical agreement of the French national ethic committee “Comité de Protection des Personnes” from Hôpital Saint Louis, Paris, France. All participants signed a written informed consent for their information to be stored in the hospital database and used for research.

#### Recorded phenotypic criteria

 Clinical, endoscopic and radiologic data were retrospectively collected and phenotypic items were validated by the referent expert gastroenterologist of the patients [2]. The involvement of ileum and colon was registered at diagnosis and at the end of follow-up. Behaviour was summarised into a binary variable following the Montreal classification (B1: non-stricturing, non-penetrating disease; non B1: stricturing and/or penetrating disease) [3]. Furthermore, information on frequency of hospitalisation, immunosuppressive medication (yes/no), surgery (yes/no) and age at diagnosis were available. Frequency of hospitalisation was categorised into never, intermediate frequency and often. Age at diagnosis was classified into lower than 17 years, from 17 to 40 years and more than 40 years according to the Montreal classification.

#### Genotyping

 Patients were genotyped for 51 earlier reported CD susceptibility SNPs using an AB17900HT Sequence detection system Illumina GoldenGate assay (Illumina, San Diego, CA) by the Centre National de Génotypage (CNG, Evry, France) and by IntegraGen (Evry, France). For each SNP the genotyping call rate was higher than 75% except for SNPs rs2631367, rs1050152 and rs2188962 which had a call rate of around 50%. Around 86% of all SNPs had a genotyping call rate higher than 0.8 and around 30% of all SNPs had a genotyping call rate higher than 0.9. For each analysed SNP, we used the following convention of nomenclature: 0 for the homozygotes carrying the frequent allele, 1 for the heterozygotes and 2 for homozygotes carrying the rare allele.

Furthermore, a subset of 450 cases was selected for additional genotyping on 30 ancestry informative markers by IntegraGen (Evry, France) to infer about population stratification and to reduce missing population information. More details on the population information can be found below. The 30 ancestry informative markers (AIMs) were selected since they were found to best discriminate with regard to F_ST_ between European, African and South-East Asian ancestries in both HapMap 2 and Human Genome Diversity Project. The call rate of the 30 ancestry markers was higher than 95%. The 450 individuals were chosen based several criteria: 1) non-European self-reported population background to maintain population diversity in the sample, 2) missing self-reported population information to reduce missing knowledge about population, 3) previous DNA quality, e.g., high call rate. 

#### Population information

On the initial data set with 845 individuals self-reported information was available regarding the continental origin (Europe, Asia and Africa ) of individuals and of their parents. Subjects were assigned to admixed origin if continental origin of father and mother differed. The majority of individuals, namely 677 (80.12%) were of European origin (see Table 1). The rest of the sample consisted of six admixed individuals (0.71%), six African individuals (0.71%), five Asian (0.59%) and 151 individuals (17.87%) with missing information (see Table 1).

**Table 1:** Population information in study sample.

	Population based on ancestry informative markers				
Self-reported population	East Asia	Europe	Sub-Saharan Africa	Missing	Total
Admixed	1	3	1	1	6
Africa	0	0	5	1	6
Asia	0	3	1	1	5
Europe	0	341	1	335	677
Missing	2	83	9	57	151
Total	3	430	17	395	845

For the subset of 450 individuals, who were genotyped on the panel of 30 ancestry informative markers, self-reported population or missing population information was replaced by population information obtained from the ancestry informative markers. This ancestry marker-based population information was derived from a PAM cluster analysis combining the 450 individuals with a HapMap 3 reference panel consisting of individuals with European, Sub-Saharan Africa or East Asian population background. The cluster analysis was performed using Euclidean distance on the 30 ancestry markers for the combined data set. All HapMap 3 individuals, who had the same population, agreed also in their PAM cluster assignment. The decision that one of the 450 individuals belonged to a given population, that is Europe, Sub-Saharan Africa or East Asia, was then based on the assigned PAM cluster. For each individual its silhouette width was determined which provides a measure of how well the individual fits into its attributed population.

Within the sample of 450 individuals, 430 subjects (95.56%) were from European ancestry (Table 1). Only three subjects (0.67%) were determined to be of East Asian ancestry, and 17 subjects (3.78%) were Sub-Saharan African. Self-reported population information was available from 356 subjects in the subset of 450 individuals and agreed with information derived from the AIMs for 346 individuals (97.19%). Four admixed individuals were attributed to the population of one of their parents as would be expected, while one admixed subject with Asian and European parents was assigned to Sub-Saharan Africa. Using the combined information from self-reports and AIMs, population information is missing in 57 subjects of the total study sample of 845 subjects (7%) instead of 151 individuals as based on the self-reports. In the following we will speak of the combined population information to refer to the joint information provided by self-reports and ancestry markers.

### Statistical analysis

Several cluster analyses were performed, i.e., model-based clustering via a latent class analysis, partitional cluster analysis using Partitioning Around Medoids algorithm and hierarchical cluster analysis using an agglomerative approach. Our focus was on commonly applied cluster analysis methods which provided an implementation in R allowing for missing data. Furthermore, the latent class analysis was chosen since it had been previously applied by Cleynen et al. [9]. Investigating in detail the performance of various different clustering analysis methods for molecular disease classification efforts was beyond the scope of this paper.

Partitioning methods identify a user specified number of clusters and assign each element to their closest cluster based on a distance measure. These methods need to be supplemented with a procedure to choose the most optimal number of clusters. In contrast, hierarchical methods involve constructing a tree of clusters in which the tree root is a single cluster containing all the observations and the leaves contain each only one observation. Agglomerative hierarchical methods (bottom-up) start with each individual defining its own cluster and merge cluster progressively according to a linkage criteria, e.g., complete-linkage, single-linkage or Ward’s method. The latter joins the two clusters that will produce the smallest increase in the pooled within-cluster variation and is based on using the squared Euclidean distance.

As opposed to the aforementioned algorithmic approaches to cluster analysis, latent class analysis is a model-based cluster analysis method that offers a variety of model selection tools and probability based classification through a posterior probability of class membership. This approach avoids the selection of an ad hoc dissimilarity or distance measure. In the following description we describe the application of latent class analysis and the use of more traditional cluster analysis methods on our available data set in separate sections. 

#### Latent Class Analysis

A latent class analysis was performed on the 51 CD associated SNPs within CD cases. Latent class analysis was applied using Latent GOLD Version 4.5 assuming binomial count variables. Missing data was handled by using the available information for each subject in the parameter estimation for the latent class model assuming that data is missing at random [23]. The number of latent classes K was determined using a bootstrap procedure and likelihood ratio (LR) testing of the null hypothesis that the population is best explained by N classes versus the alternative hypothesis with N + 1 classes. Per latent class model, 500 bootstrap samples were generated. Individuals were assigned into their class based on the highest membership probability obtained from the latent class analysis. Influential SNPs for a given class within the final latent class model were assessed via Z-scores as provided by the Latent GOLD software. All formal testing was performed at a nominal significance level of 0.05.

#### Principal component analysis

The available panel of 30 ancestry informative markers were submitted to a principal components analysis in the R-package *snpStats* version 1.8.1 and R-version 2.15.3 [24]. Prior to the principal component computation, the matrix *G*=(*g*_*ij*_)_*i*=1,...,*n*,*j*=1,..,*m*_ containing the genotypes *g*_*ij*_ (=0, 1 or 2) for an individual *i (i=1, ... , n*) at marker *j* (*j*=1, ..., *m*) was standardised. Standardisation was obtained by subtracting the column mean 2*p*_*j*_ from each column in *G* and by dividing each column by its standard deviation$\sqrt{2p_{j}(1-p_{j})}$, where *p*_*j*_ is the estimated minor allele frequency of marker *j* [25]. Missing genotypes were replaced by zeros in the standardised matrix. Following Price et al. [26], a correction for population stratification was then applied on the genotype matrix *G* by projecting the genotypes onto the space orthogonal to the space spanned by the principal components:

$$G^{'}=(I_{n}-A{\left(A^{T}A\right)}^{-1}A^{T})G,$$ (1)

where *I*_*n*_ is the identity matrix of size *n* and *A* is a *n*×*k*matrix containing (a subset of ) *k* principal components. Missing genotypes in *G* are not taken into account for calculation of the population stratification adjusted genotypes *G* ^'^ and are set to missing in *G* ^'^ as well.

#### Cluster analysis

All 450 individuals were subsequently clustered in R version 2.15.3 based on their population stratification adjusted genotypes g_ij_, using either hierarchical agglomerative cluster analysis as implemented in the *hclust* function or Partitioning Around Medoids (PAM) clustering algorithm via the *pamk* function [27]. No model-based cluster analysis was performed on the continuous population-adjusted SNP data since this analysis would have been based on the assumption of a Gaussian distribution for the variables. This assumption was not justified here. The PAM algorithm was applied using Euclidean distance and the average silhouette width to determine the optimal number of clusters [28]. The hierarchical cluster analysis was applied using Ward’s minimum variance criterion and squared Euclidean distance. For the hierarchical cluster analysis, the agglomeration criterion values, which correspond here to the distance between the two clusters joined at a given agglomeration step, were considered to determine the number of clusters. The distance between two clusters is proportional to the distance between their two centroids. A strong increase in the agglomeration criterion indicates that two dissimilar clusters are being joined and that the agglomeration can be stopped at this step. The strength of increase was evaluated by studying the percentage change in agglomeration criterion when going from N + 1 clusters to N clusters. For reasons of parsimony, we studied the percentage change in the agglomeration criterion over the last ten agglomeration steps. Both functions *pamk* and *hclust* were able to handle missing data by considering only available data per individual. When SNPs were excluded in the calculation of the pairwise distance between two subjects, the distance was scaled up proportionally to the number of SNPs used.

Furthermore, we applied the PAM clustering algorithm and hierarchical cluster analysis directly on the population unadjusted SNP variables of the 450 individuals to enable a more direct comparison between the results on the adjusted and non-adjusted SNP data by using similar methodology. As before on the population adjusted SNPs, the Euclidean distance was applied for the PAM algorithm while the squared Euclidean distance and Ward’s minimum variance criterion were used for the hierarchical cluster analysis. 

#### Association testing of genetic-based subgroups and clinical subphenotypes

Association tests between clinical subphenotypes and identified genetic-based groups were performed in R 2.15.3 using univariate Fisher’s exact tests or Pearson’s chi-squared tests (with or without bootstrap sampling) depending on which method was most appropriate. The similarity of the various groupings obtained by the cluster analysis methods were compared pairwise to each other using the adjusted Rand index (ARI). The adjusted Rand index is bounded above by 1, where 1 indicates perfect agreement between two groupings. Values of the adjusted Rand Index above 0.9 can be seen as excellent agreement, values above 0.8 as good agreement and values greater than 0.65 can be considered as moderate agreement, while values below 0.65 show poor agreement [29].

## Results

### Latent class analysis using population unadjusted SNP data

Latent class analysis and bootstrap LR test on 51 CD-associated SNPs identified nine classes in the 845 individuals. A subset of 475 individuals (i.e., 56% ) had a class membership probability higher than 0.9 for their assigned class and hence a reliable assignment to one specific patient class could be made for these individuals. For a total of 186 patients (22%) the maximum class membership probability was lower than 0.6. Nevertheless, these patients were assigned to their most probable patient subgroup.

Table 2 shows the p-values of the association tests between the classes and available clinical subphenotypes. When using the latent class analysis, significant results (p < 0.05) were obtained for the variables indicating terminal ileum disease location. The classes differed thus most strongly with regard to whether or not disease location at diagnosis was terminal ileum but results were no longer significant after Bonferroni correction for multiple testing. Class 6 and 8 contained the highest and lowest proportion of individuals with terminal ileum disease location, respectively (Table 3). In the previous statement, class 5 and 9 are not taken into account since these classes contained many individuals with missing information on the clinical subphenotypes. Table 3 gives a complete characterization of the CD patients in our data according to clinical subphenotypes and latent classes. 

**Table 2:** P-values for association testing of clusterings with clinical subphenotypes.

		Population unadjusted data			Population adjusted data	
		Latent classes	PAM clusters	Hierarchical clusters	PAM clusters	Hierarchical clusters
*Age at diagnosis (Montreal)*		0.2966	0.6213	0.9644	0.3799	0.3925
*Disease location*						
	Terminal ileum at diagnosis	0.0094	0.2539	0.5280	0.9124	0.8423
	Terminal ileum at follow-up	0.0291	0.0691	0.1674	0.8993	1.0000
	Colon at diagnosis	0.9965	0.4964	0.3616	0.5751	0.0719
	Colon at follow-up	0.9774	0.2269	0.0636	0.8984	0.0670
*Disease behaviour*						
	Behaviour B1 at diagnosis	0.0840	0.8815	0.7644	0.0569	0.6620
	Behaviour B1 at follow up	0.5800	0.1412	0.5824	0.6860	0.7402
*Frequency of hospitalisation*		0.4205	0.2270	0.2457	0.4672	0.0538
*Immunosuppressive medicaments*		0.2318	0.9491	0.6560	0.8194	0.7629
*Surgery*		0.4659	0.2501	0.9623	0.3739	0.9447

**Table 3:** Characteristics of individuals in overall data set and in latent classes.

				Class 1	Class 2	Class 3	Class 4	Class 5	Class 6	Class 7	Class 8	Class 9	Total
*Subjects*				407	163	136	33	32	24	18	17	15	845
*Age at diagnosis*													
	<17			94 (24%)	53 (33%)	45 (33%)	5 (15%)	0 (0%)	9 (38%)	5 (28%)	8 (47%)	0 (0%)	219 (28%)
	17-40			276 (69%)	98 (61%)	79 (59%)	27 (82%)	1 (100%)	14 (58%)	11 (61%)	8 (47%)	1 (100%)	515 (65%)
	>=40			30 (8%)	10 (6%)	11 (8%)	1 (3%)	0 (0%)	1 (4%)	2 (11%)	1 (6%)	0 (0%)	56 (7%)
*Disease location*													
	Terminal ileum at diagnosis			251 (65%)	122 (77%)	99 (75%)	22 (67%)	1 (100%)	22 (92%)	14 (82%)	8 (53%)	1 (100%)	540 (71%)
	Terminal ileum at follow-up			307 (77%)	139 (86%)	116 (85%)	25 (76%)	1 (100%)	24 (100%)	16 (89%)	12 (71%)	1 (100%)	641 (81%)
	Colon at diagnosis			286 (74%)	115 (73%)	95 (73%)	25 (78%)	1 (100%)	17 (71%)	13 (76%)	13 (76%)	1 (100%)	566 (74%)
	Colon at follow-up			330 (83%)	129 (80%)	112 (83%)	26 (81%)	1 (100%)	20 (83%)	15 (88%)	14 (82%)	1 (100%)	648 (82%)
*Disease behaviour*													
	B1 at diagnosis			313 (80%)	120 (75%)	102 (77%)	26 (79%)	0 (0%)	14 (61%)	12 (67%)	16 (94%)	1 (100%)	604 (78%)
	B1 at follow-up			187 (48%)	67 (42%)	53 (40%)	14 (42%)	0 (0%)	8 (33%)	8 (44%)	7 (41%)	1 (100%)	345 (45%)
*Frequency of hospitalisation*													
	Never			62 (16%)	21 (13%)	19 (15%)	6 (19%)	0 (0%)	4 (17%)	3 (18%)	3 (18%)	0 (0%)	118 (15%)
	Intermediate			286 (74%)	125 (79%)	99 (76%)	23 (72%)	1 (100%)	19 (79%)	13 (76%)	10 (59%)	0 (0%)	576 (75%)
	Often			41 (11%)	13 (8%)	12 (9%)	3 (9%)	0 (0%)	1 (4%)	1 (6%)	4 (24%)	1 (100%)	76 (10%)
*Immunosuppressive medicaments*				297 (74%)	125 (77%)	102 (75%)	23 (70%)	1 (100%)	14 (58%)	9 (50%)	13 (76%)	1 (100%)	585 (74%)
*Surgery*				196 (49%)	87 (54%)	69 (51%)	17 (52%)	1 (100%)	17 (71%)	11 (61%)	9 (53%)	0 (0%)	407 (51%)

Class 5 contains 31 individuals with missing information on all clinical characteristics. Class 9 contains 14 individuals with missing information on all clinical characteristics. For the other classes, the number of individuals with missing information differs between the clinical characteristics.

Using the Z-scores obtained for each SNP variable and each class within Latent GOLD, four SNPs had Z-scores significantly different from zero for class 6 and were thus characteristic for this class: rs2066847 (NOD2), rs2241880 (ATG16L1) and rs17582416 (intergenic region on 10p11) and rs744166 (STAT3). Restricting to class 6 individuals, the minor alleles of these SNPs showed the following frequencies based on the latent class model: 0.997 for rs2066847 with minor allele C (highest allele frequency of all classes), 0.1331 for rs2241880 with minor allele G (lowest allele frequency of all classes), 0.1907 for rs17582416 with minor allele G (lowest allele frequency of all classes) and 0.5405 for rs744166 with minor allele G (highest allele frequency of all classes). 

The distribution of population strata (according to the combined information) over the identified nine classes is presented in Table 4. These results show that that all classes, except class 8, were mostly populated by individuals with a European background. Although only 17 of 845 patients (2.01%) were assigned to class 8, the majority (70.59%) of these 17 individuals were from Sub-Saharan Africa or Africa. The association between the populations and the classes was significant (Pearson’s chi-squared test with 99999 bootstrap samples, p = 0.00001). The following two findings about two of the four Europeans in class 8 seem to be in line with the mainly African nature of class 8. Firstly, one individual in class 8, which was assigned to Europe based on the AIMs and thus represented as European in Table 4, was admixed with a European and African parent based on self-report. Secondly, one European individual in class 8 had only a class membership probability of 0.61, which does not indicate a strong class membership. 

**Table 4:** Distribution of populations over clusters obtained by latent class analysis.

	Admixed	Africa	Asia	East Asia	Europe	Sub-Saharan Africa	Missing	Total
Class 1	0 (0%)	0 (0%)	1 (0.25%)	1 (0.25%)	370 (90.91%)	4 (0.98%)	31 (7.62%)	407 (100%)
Class 2	1 (0.61%)	0 (0%)	0 (0%)	1 (0.61%)	146 (89.57%)	2 (1.23%)	13 (7.98%)	163 (100%)
Class 3	0 (0%)	0 (0%)	0 (0%)	0 (0%)	134 (98.53%)	0 (0%)	2 (1.47%)	136 (100%)
Class 4	0 (0%)	0 (0%)	0 (0%)	0 (0%)	31 (93.94%)	0 (0%)	2 (6.06%)	33 (100%)
Class 5	0 (0%)	0 (0%)	0 (0%)	1 (3.13%)	24 (75%)	0 (0%)	7 (21.88%)	32 (100%)
Class 6	0 (0%)	0 (0%)	0 (0%)	0 (0%)	24 (100%)	0 (0%)	0 (0%)	24 (100%)
Class 7	0 (0%)	0 (0%)	0 (0%)	0 (0%)	18 (100%)	0 (0%)	0 (0%)	18 (100%)
Class 8	0 (0%)	1 (5.88%)	0 (0%)	0 (0%)	4 (23.53%)	11 (64.71%)	1 (5.88%)	17 (100%)
Class 9	0 (0%)	0 (0%)	0 (0%)	0 (0%)	14 (93.33%)	0 (0%)	1 (6.67%)	15 (100%)
Total	1 (0.12%)	1 (0.12%)	1 (0.12%)	3 (0.36%)	765 (90.53%)	17 (2.01%)	57 (6.75%)	845 (100%)

### Principal component analysis

**Figure 1:**
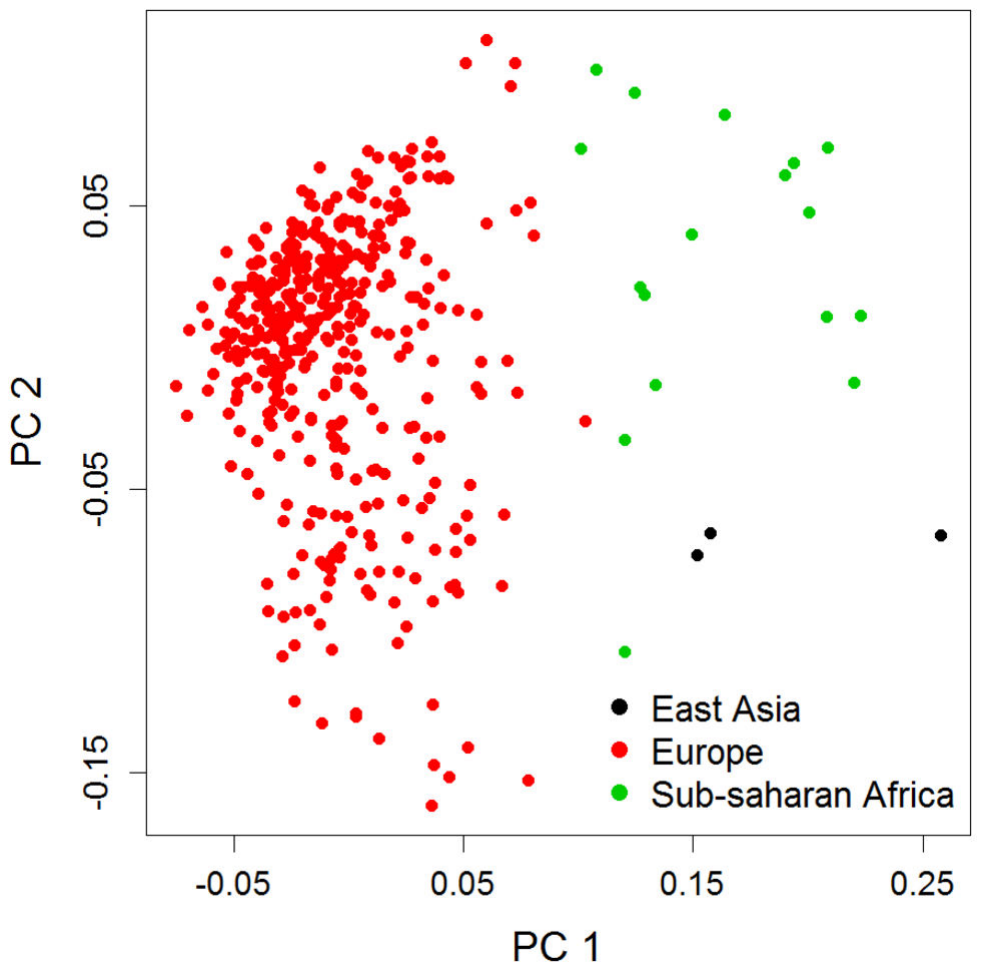
Principal components based on 30 ancestry informative markers for 450 individuals.

Figure 1 shows a scatterplot of 450 CD patients according to the first two principal axes of genetic variation based on 30 ancestry SNPs. These two principal components PC1 and PC2 explained 18% of the variation captured by the 30 ancestry SNPs and were considered in the next section to correct for population stratification in the three ancestry-marker derived populations (East Asia, Sub-Saharan Africa, and Europe). Whereas PC1 differentiated between Europe and Non-Europe, PC2 arguably seemed to discriminate between Africa and Asia. There was one strong outlier, who despite being attributed to Sub-Saharan Africa based on the set of 30 AIMs, had a PC2 value lower than all the East Asian individuals. The self-reported origin of this individual was Africa and its silhouette width for the assignment into Sub-Saharan Africa was 0.06. This low silhouette width and the self-reported information indicate that the subject is perhaps from Africa but might be closer to North Africa than to Sub-Saharan Africa in its genetic background. 

### Cluster analysis of CD patients using population adjusted und unadjusted SNP data

Using the PAM clustering algorithm on the population adjusted SNP data within the subset of 450 individuals only two clusters were found based on the silhouette width. The average silhouette width was highest for two clusters with a value of 0.0112. However, such a low value for the average silhouette width indicates an extremely low to non-existing structure in the data. Individual silhouette widths for clusters varied between -0.05 and 0.06 for cluster 1 and between -0.03 and 0.32 for cluster 2. In general, a value close to zero indicates that an individual is at the border between two clusters while a value close to -1 indicates that the individual is better attributed to a neighbouring cluster. No significant association between any clinical subphenotype and the PAM clustering was found based on Fisher’s exact test (see Table 2). 

**Figure 2:**
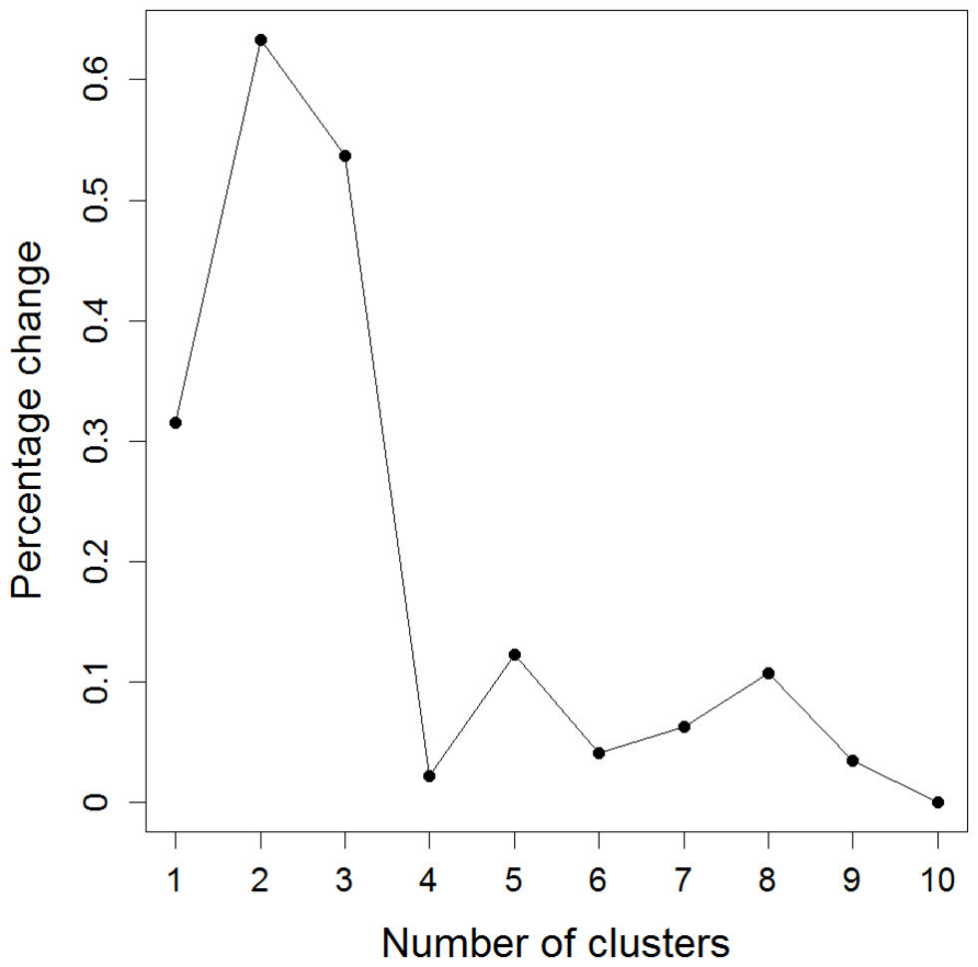
Percentage change in agglomeration criterion (hierarchical cluster analysis using population adjusted SNPs).

Using the hierarchical cluster analysis and population adjusted SNP data, three clusters were detected in the 450 patients after visual inspection of the percentage change in the agglomeration criterion as can be seen from Figure 2. The percentage change was highest when joining three to two clusters indicating that two quite dissimilar clusters were being joined. Therefore, the agglomeration could better be stopped at this stage. Table 2 shows that frequency of hospitalisation, colon at diagnosis and colon at follow-up were nearly significant based on Fisher’s exact test (p = 0.0538, p = 0.0719 and p = 0.0670, respectively). In Table 5 an overview of the distribution of phenotypic characteristics over the hierarchical clusters and in the subset of 450 individuals is presented. Table 5 shows that cluster 3 contained a lower percentage of individuals with colon as disease location (33% at diagnosis and 50% at follow-up) than the other clusters. Notably, neither the PAM clusters nor the hierarchical clusters were significantly associated with the population structure (Fisher’s exact test, respectively p = 0.1392 and p = 0.4608 for PAM and hierarchical agglomerative clustering). 

**Table 5:** Characteristics of individuals in subset of 450 individuals and in hierarchical clusters based on population adjusted SNPs.

		Cluster 1	Cluster 2	Cluster 3	Total
*Subjects*		309	102	39	450
*Age at diagnosis*					
	<17	90 (29%)	25 (25%)	0 (0%)	115 (28%)
	17-40	194 (63%)	67 (66%)	6 (100%)	267 (64%)
	>=40	22 (7%)	10 (10%)	0 (0%)	32 (8%)
*Disease location*					
	Terminal ileum at diagnosis	209 (71%)	68 (69%)	4 (67%)	281 (70%)
	Terminal ileum at follow-up	249 (81%)	83 (81%)	5 (83%)	337 (81%)
	Colon at diagnosis	219 (74%)	69 (70%)	2 (33%)	290 (72%)
	Colon at follow-up	252 (83%)	79 (78%)	3 (50%)	334 (81%)
*Disease behaviour*					
	B1 at diagnosis	226 (76%)	79 (79%)	4 (67%)	309 (77%)
	B1 at follow-up	124 (42%)	46 (46%)	2 (33%)	172 (43%)
*Frequency of hospitalisation*					
	Never	41 (14%)	11 (11%)	1 (17%)	53 (13%)
	Intermediate	220 (76%)	80 (80%)	2 (33%)	302 (76%)
	Often	30 (10%)	9 (9%)	3 (50%)	42 (11%)
*Immunosuppressive medicaments*		229 (75%)	78 (76%)	4 (67%)	311 (75%)
*Surgery*		169 (55%)	55 (54%)	3 (50%)	227 (55%)

Cluster 1 contains three individuals with missing information on all clinical characteristics. Cluster 3 contains 33 individuals with information missing on all clinical characteristics. For cluster 2 each individual provides information for at least one clinical characteristic.

Furthermore for comparison with the results above, we applied PAM and hierarchical cluster analysis on the 51 CD associated SNPs without adjustment for population stratification within the subset of 450 individuals. For the PAM clustering, the average silhouette width had a maximum value of 0.03 for three clusters. This indicates that there is limited structure in the data based on the PAM clustering. Fisher’s exact test showed a significant association between the ethnicity based population strata (East Asia, Sub-Saharan Africa, and Europe) and the PAM clusters (p = 0.0002). The majority of Sub-Saharan Africans in the data (65%) belonged to PAM cluster 3 although PAM cluster 3 only represented 21% of all 450 individuals. 

**Figure 3:**
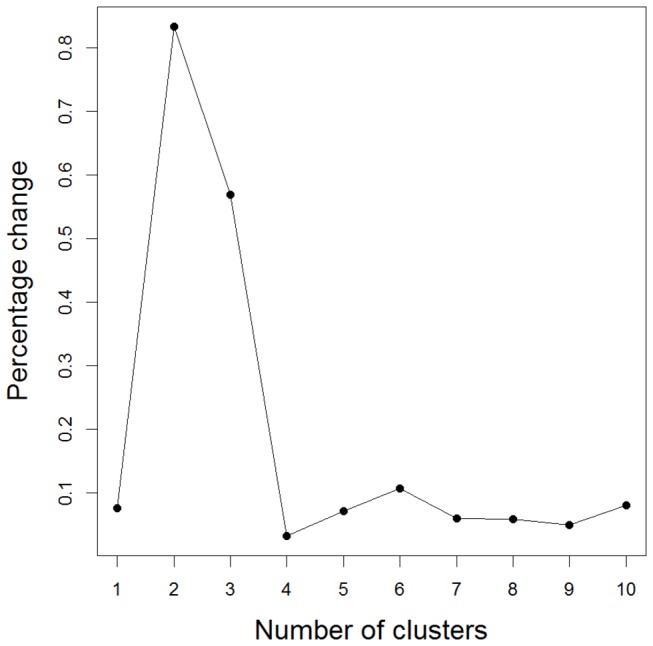
Percentage change in agglomeration criterion (hierarchical cluster analysis using population unadjusted SNP markers).

For the hierarchical agglomerative clustering of patients, the percentage change in the Ward agglomeration criterion, i.e., the squared Euclidean distance between the two clusters joined at this step, was the highest when joining three to two clusters (see Figure 3). Detailed inspection of the 3-cluster solution highlights a significant association between the three populations and the three hierarchical clusters using Fisher’s exact test (p = 0.0005). In particular, 11 of the 17 Sub-Saharan African individuals (65%) belonged to hierarchical cluster 2, although this cluster contained only 98 of 450 individuals (22%). No significant association could be found between the PAM clustering or hierarchical clustering for any of the subphenotypes (see Table 2).

The adjusted Rand indexes were between 0.04 and 0.54 thus indicating a poor agreement of the different groupings with each other (see Table 6). It seemed that the groupings based on the unadjusted SNP agreed better with each other (ARIs between 0.23 and 0.49) than with the groupings based on the ancestry adjusted SNPs (ARIs between 0.04 and 0.23 except for the two hierarchical clusterings which reached an ARI of 0.54). Notably, the PAM and hierarchical clustering based on the adjusted SNP markers had a very low ARI of 0.04. 

**Table 6:** Adjusted Rand Indexes between latent class analysis (LCA), PAM clustering and hierarchical clustering (HC) (using population unadjusted or adjusted SNP data).

		Unadjusted			Adjusted	
		LCA	PAM	HC	PAM	HC
Unadjusted	LCA		0.49	0.30	0.12	0.23
	PAM			0.23	0.20	0.13
	HC				0.04	0.54
Adjusted	PAM					0.04
	HC					

## Discussion

Crohn’s disease is a complex disorder with heterogeneity in disease symptoms, characteristics and development between patients. Due to the validated genetic background of Crohn’s disease it is of interest to assess the extent by which disease susceptible markers can help to identify disease subtypes or patient subgroups. In a first study, Cleynen et al. [9] applied latent class analysis to molecularly reclassify CD patients based on a selection of 46 markers identified from GWA studies on CD and/or meta-analysis of these [4]. However, they did not consider the potential interference between molecular reclassification and population substructure. In this paper, we proposed principal components to correct SNPs for population stratification prior to cluster analysis and showed that quite different results can be obtained while adjusting for or ignoring population substructure. 

It was seen that the adjusted Rand indexes were low thus indicating little overlap between the different cluster analysis methods even when applied on the same data source. However, this might be expected since different cluster analysis methods use different criteria for cluster formation and can thus lead to different results [30]. Furthermore, the latent class analysis was performed on the full study sample while the other cluster analysis methods were applied only on the subset of 450 individuals for comparison among each other. Another reason for the little overlap in the groupings could be that there was inherently little structure in the data as indicated by the low silhouette widths for the PAM algorithm and the low percentage of individuals with high maximum class membership probability. 

The number of clusters varied also between the methods although the PAM algorithm with average silhouette width and the hierarchical cluster analysis with percent change in agglomeration criterion seemed to agree in the fact that a lower number of clusters was optimal. It should be mentioned that using a Bayesian information criterion to determine the optimal number of classes for the latent class analysis within the full sample also led to only three clusters. For future research it will be beneficial to perform a more detailed comparison of different cluster analysis methods with regard to the identification of genetic-based subgroups on larger data sets. However, the focus in this paper was on the correction for population stratification when identifying genetic-based disease subgroups and not on the comparison of different cluster analysis methods.

Association tests with the clusterings – before and after correction for population using the principal components – indeed revealed that, before correction, groupings were significantly associated with population structure while after correction no significant association could be detected. All analysis methods on the population unadjusted data seemed to have one group with a mainly African background. This shows that, as expected, cluster analysis based on SNP data can be confounded by population stratification. However, it was also seen that the suggested approach of using the principal components to obtain population adjusted SNP data solved the issue of population stratification. 

Despite our observation that latent classes of patients were likely to be confounded by population stratification, the significant associations found between terminal ileum at diagnosis and the latent classes were interesting from several perspectives. Relevant SNPs for the latent class with the highest percentage of terminal ileum at diagnosis were SNPs rs2066847 (NOD2), rs2241880 (ATG16L1), rs17582416 (intergenic region on 10p11) and rs744166 (STAT3). Notably, NOD2 has repeatedly been shown to be associated with terminal ileum as disease location [6-8], and rs2241880 has also been described as being associated with ileal disease location [31,32]. Furthermore, two of these four SNPs, namely rs2066847 and SNP rs2241880 are part of one pathway, the autophagy pathway, previously reported to play a role in CD disease pathogenesis [33]. Jung et al. [2] performed a genotype/phenotype analysis using logistic regression on a slightly different version of the data used in this paper and could also detect an association of NOD2 variants with ileal disease location. This agreement with previous results might be seen as a confirmation that cluster analysis can be applied to detect subphenotype associated variants.

When correcting for population stratification, evidence for a significant association between terminal ileum and the clusterings no longer existed, irrespective of whether hierarchical or PAM algorithms were used. In contrast, the hierarchical clustering showed an almost significant association for disease location in the colon. None of the associations reported here is however significant when corrected for multiple testing using Bonferroni correction. Note that ignoring population strata for the hierarchical or PAM cluster analysis were also unable to establish significant associations between genetic-based subgroups.

One explanation why an interesting result might have been found when not correcting for confounding by population stratification is the following. While risk alleles for CD at the NOD2 locus are frequent in Caucasian populations, these risk alleles are less frequent in African or Asian populations [34,35]. Not only might there be differences with regard to genotype between races but also differences with regard to clinical subphenotype, e.g., it seems that African Americans have less frequently ileal disease location than Caucasians [36]. These different prevalences for subphenotypes might be caused by different allele frequencies due to ancestry. Similarly, Myles et al. [18] imply that highly differentiated allele frequencies between populations may lead to differences in disease prevalence between populations. In such a case correcting for population stratification will actually unwantedly remove differences in allele frequencies so that subphenotypes associated with these alleles cannot be detected anymore.

For future subphenotype analysis, it might thus be recommendable to focus on populations with a similar genetic background, e.g., Europeans, for a cluster analysis on genetic markers. However, the main scope of this paper was not to detect genetic-based groups associated with subphenotypes but to study the effect of population stratification on the detection of genetic-based subgroups and to describe the benefits and potential limitations of principal components to correct for population stratification. The use of principal components in a regression framework is perhaps the most popular method to correct for population stratification in genetic association studies. Advantages of using principal components instead of other methods to correct for population stratification are computational efficiency and ease of use, although the selection of principal components to include in the analysis is often based on heuristics.

Other common methods to control for population stratification in association studies are genomic control [37] and the program STRUCTURE [15] . However, genomic control is not applicable in our context since it directly corrects the potentially inflated test statistics from an association study [37]. Using STRUCTURE we would be able to separate our subjects into several subpopulations and subsequently perform a cluster analysis on each subpopulation. However, such a procedure would lead to a reduction of the sample size for each analysis and thus a loss of power. Furthermore, this approach would ignore a clinal pattern of genetic variation in the data which can be captured by principal components. Human genetic variation has been argued to be better described by clinal patterns than by partition into distinct subpopulations [38].

In this study, we used ancestry informative markers to calculate the principal components enabling us to separate continental populations. This is a useful approach for candidate marker studies where information on population stratification cannot be obtained from a genome-wide set of markers. The additional sampling of ancestry markers is then a simple and cost-effective alternative to detect population structure.

There are several reasons why we might not have been able to find a strong association between the subphenotypes and the detected clusters. Firstly, efforts should be undertaken to repeat the analysis strategies on a much larger cohort to improve power. Currently, analysis is ongoing to perform such strategies on data consisting of ~ 19,000 Crohn’s disease patients from The International IBD Genetics Consortium. Secondly, using a larger number of CD associated variants, e.g., all currently known 110 CD associated genetic markers, might also improve the classification results. Thirdly, Cleynen et al. [9] and Jung et al. [2] hypothesized that different genetic variants might be relevant for discrimination between subphenotypes than for discrimination between cases and controls. Performing a genome-wide cluster analysis has the advantage that such variants can be discovered. Disadvantages of a genome-wide cluster analysis are the increased computation time and the difficulty to find the set of relevant genetic markers for subphenotypes within a huge set of uninformative genetic markers. A future topic of research could be to investigate the possibility of genome-wide cluster analysis despite the massive computational effort and to develop suitable methodology. 

Further in-depth investigations are needed to prove the suggested methodology based on simulated data. Simulation studies could generate data with population structure using the Balding-Nichols model [39] and additional structure due to gene variants associated with subphenotypes. It is then possible to evaluate the performance of several methods to correct for population stratification, prior to or during different cluster analyses, on data with known substructures. Such a simulation study can also be useful to provide recommendations on which cluster analysis methods would be most efficient in discovering substructure based on genetic-based patient subgroups after correction for population stratification. 

To conclude, we showed that principal components can be used to correct SNP data for population stratification before performing a cluster analysis. While our focus has been on Crohn’s disease, the presented methodology can be applied to any complex heterogeneous disease. Furthermore, our aim was to explore phenotypic variability by relating the detected genetic-based groups to clinical subphenotypes, but Thornton-Wells et al. [40] and Thornton-Wells et al. [11] suggested using cluster analysis to unravel various other forms of heterogeneity, e.g., trait heterogeneity and genetic heterogeneity as well. Thus, the presented methodology to correct for population stratification in cluster analysis of SNP data might also prove useful for detection of other factors of heterogeneity in complex human diseases.
